# Telehealth Listening Visits for emotionally distressed mothers of hospitalized newborns: Proof-of-concept

**DOI:** 10.3389/fpsyt.2022.1032419

**Published:** 2022-12-07

**Authors:** Lisa S. Segre, Jennifer E. McCabe, Rebecca Chuffo Davila, Stephan Arndt

**Affiliations:** ^1^College of Nursing, The University of Iowa, Iowa City, IA, United States; ^2^Department of Psychology, Western Washington University, Bellingham, WA, United States; ^3^University of Iowa Stead Family Children’s Hospital, Iowa City, IA, United States; ^4^Department of Psychiatry, The University of Iowa, Iowa City, IA, United States

**Keywords:** Listening Visits, neonatal intensive care unit (NICU), maternal emotional distress, NICU nurse, telehealth, task shifting, nurse-delivered counseling

## Abstract

**Problem:**

Newborn admission to the neonatal intensive care unit (NICU) is stressful. Yet in clinical practice, at best, NICU mothers are screened for depression and if indicated, referred to a mental-health specialist. At worst, no action is taken. Listening Visits, an empirically supported nurse-delivered intervention addressing emotional distress, can be conveniently provided by a NICU nurse. Prompted by the need to minimize face-to-face contacts during the pandemic, the primary purpose of this small pilot trial was to assess the feasibility of having nurses provide Listening Visits to NICU mothers over Zoom. Secondarily, we assessed pre-to post-Listening Visits depression symptom scores as a preliminary evaluation of the effectiveness of this type of support.

**Materials and methods:**

Nine NICU mothers with mildly to moderately severe depression symptoms received up to six Listening Visits sessions from a NICU nurse via Zoom. Participants completed the Inventory Depression and Anxiety Symptoms-General Depression scale (IDAS-GD) at study entry and 4- and 8-weeks post enrollment. They completed the Client Satisfaction Questionnaire (CSQ) at the 8-week assessment.

**Results:**

Using an intent-to-treat approach, the effect of time from the mixed model analysis of IDAS-GD was statistically significant (*F*(2,26) = 10.50, *p* < 0.001), indicating improvement in IDAS-GD scores from baseline to follow-up. The average CSQ score was high (*M* = 29.0, SD = 3.3), with 75% of participants rating the quality of help they received as “excellent”.

**Discussion:**

In this pilot trial, we learned it is feasible to provide Listening Visits over Zoom, that this mode of delivery is associated with a significant decrease in depression symptom scores, and that women value this approach.

**Clinical trial registration:**

[https://clinicaltrials.gov/], identifier [#201805961].

## Introduction

Having one’s newborn admitted to a neonatal intensive care unit (NICU) is a quintessential distressing event. Accordingly, mothers of hospitalized newborns are at increased risk for depression (1), with 33% of these mothers experiencing suicidal thoughts, well above the 2–9% reported for mothers of non-hospitalized newborns (2, 3). Setting suicidal ideation aside, the results of discharge readiness studies suggest that the diminished parenting that is associated with maternal emotional distress may also interfere with a family’s readiness for discharge, which is determined not only by the newborn’s clinical condition but also by parental confidence and competence (4). Prior work has established that maternal distress negatively affects the social and emotional development of both term infants (5, 6) and premature infants (7–10). And while mothers of premature infants engage in more maternal interactions than mothers of term infants, distressed mothers engage in fewer maternal interactions than non-distressed mothers regardless of infant birth status (11). This effect persists even 2 years later (12).

Despite this pressing need for emotional support, NICU mothers are, generally speaking, at best screened for depression and referred to a mental health specialist if appropriate; in the worst case, no screening or referral takes place. The dichotomy between full-on versus no mental health care represents a strikingly inadequate response for mothers dealing with a normative reaction to a stressful event. One solution is Listening Visits, an evidence-based emotional support intervention conveniently delivered at the newborn’s point-of-care by a nurse, a trusted professional who is coincidentally also medically knowledgeable about hospitalized newborns.

In the early 1980s a British health visitor (home visiting nurses in the UK’s National Health Service) developed Listening Visits, a nurse-delivered intervention to support depressed postpartum women (13). Based on Roger’s client centered therapy, the rationale for this intervention is that the expression of feelings and consistent social support helps reduce depression and anxiety symptoms (14). The key components of Listening Visits include empathic listening, to fully understand a woman’s situation, and problem solving, to work collaboratively with a woman to generate specific solutions. The first randomized controlled trial evaluation found that 69% of Listening Visit recipients no longer met criteria for major depression, compared to only 38% of those receiving usual care (13). The efficacy of Listening Visits delivered by home visiting nurses has been empirically demonstrated in subsequent randomized controlled trials in the UK (15, 16), Sweden (17), Norway (18), and the United States (19). A meta-analysis of these trials found a moderate effect size for Listening Visits as provided by home visitors to depressed postpartum women of full term infants (20).

Having NICU nurses deliver Listening Visits to emotionally distressed women represents an innovative model of care with the promise of delivering much needed emotional support for mothers of hospitalized newborns (21). Two proof of concept trials provide evidence for the feasibility, safety, acceptability, and effectiveness of Listening Visits when delivered by a neonatal nurse practitioner to women with mildly to moderately severe depression symptoms (22), as well as by bachelor’s degree NICU staff nurses (23). In both of these trials the visits were conducted in person, on the NICU, every 2–3 days. This schedule represents a change from the once-a-week visits used in home visiting settings. This adapted schedule was deemed necessary for two reasons. The health of a hospitalized newborns can vary significantly from day to day and a woman may need support more than once a week. And to receive all six sessions before newborn discharge, a shorter period is required. Results of both trials were promising. Listening Visits resulted in decreased depression scores and proved feasible for nurses to implement in the NICU, in the sense that the associated workload for nurses was manageable, there were no instances of significant clinical adverse events, and it was also possible to find a suitably private hospital location for the sessions themselves. On the other hand, in-person Listening Visit sessions were not always accessible for mothers with work, family, or geographic constraints that kept them from spending time with their newborn at the hospital. Even among participants who received Listening Visits, it was sometimes difficult for them to meet twice per week, resulting in some newborns being discharged before the mother could participate in all six sessions. Thus, virtual Listening Visits could make this form of support available to more women and allow more women to complete their sessions at the requisite twice per week pace.

In March 2020, widespread suspension of hospital based clinical trials because of the COVID-19 pandemic caused our pilot RCT to close with 45 of 50 women enrolled (23). At the same time, increased availability, and familiarity with the Zoom virtual conferencing platform, coupled with the increased flexibility demonstrated by many IRBs in allowing researchers to make use of this platform, prompted us to explore the feasibility of having NICU nurses provide Listening Visits via Zoom. We quickly sought IRB approval and assembled a small pilot sample of mildly to moderately depressed mothers of hospitalized newborns. The primary aim of this pilot trial was to assess whether it was practicably feasible for RNs and women to connect via Zoom for Listening Visits. Secondarily, we collected preliminary data on change in depression symptoms as well as women’s satisfaction with this mode of treatment.

## Materials and methods

### Study design and ethics

This study utilized an open pilot design which was approved by the University’s IRB and registered with ClinicalTrials.gov (#201805961).

### Participants

**FIGURE 1:**
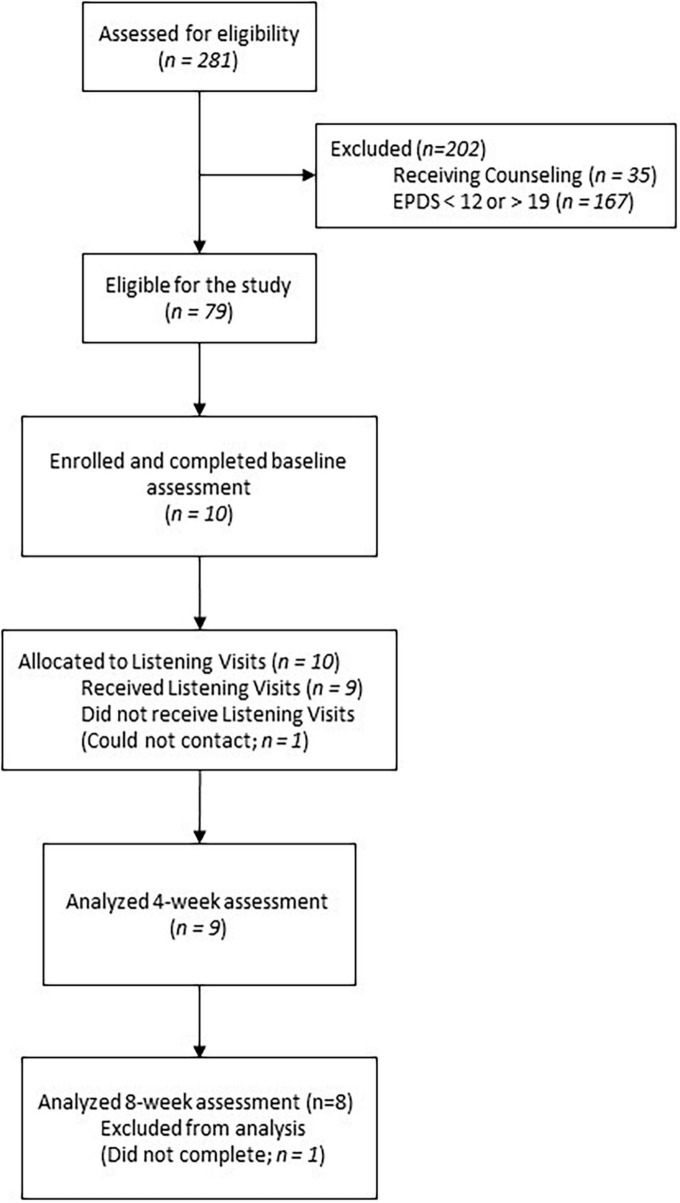
Consort flow diagram.

From February 2021 through May 2022, 79 women met the demographic and clinical eligibility criteria, and nine consented and received Listening Visits (Figure 1). To be demographically eligible women had to be 18 years or older, have a currently hospitalized newborn, and could not be receiving counseling. To be clinically eligible, women needed to have mildly to moderately severe depression symptoms on the Edinburgh Postnatal Depression Scale [EPDS; (24)], which was operationalized as a score of 12–19 inclusive (25). As indicated in Table 1, the nine participants had an average age of 27.8 years (SD = 3.5). They were predominantly White (77.8%), with none identifying as Hispanic/Latina. Most participants had at least some college education (66.6%) and reported annual incomes of $50,000 or more (66.6%).

**TABLE 1:** Demographics and Listening Visits session information.

Variable	Mean (SD)
Age	27.78 (3.46)
Hours per week worked for pay	2.67 (0.87)
Sessions completed by 4 weeks	3.78 (1.86)
Sessions completed by 8 weeks	4.56 (1.51)

**Variable**	**Frequency (%)**

**Race/Ethnicity**	
White	7 (77.8%)
Black or African American	2 (22.2%)
Hispanic/Latina	0 (0%)
**Highest education**	
High school diploma/GED	3 (33.3%)
Associate degree	1 (11.1%)
Bachelor’s degree	3 (33.3%)
Master’s degree	2 (22.2%)
**Income**	
Lower than $25,000	3 (33.3%)
50,000–$74,999	1 (11.1%)
75,000-$99,999	3 (33.3%)
100,00 or higher	2 (22.2%)
**Sessions completed by 4 weeks**	
Two	4 (44.4%)
Four	2 (22.2%)
Six	3 (33.3%)
**Sessions completed by 8 weeks**	
Two	1 (11.1%)
Three	1 (11.1%)
Four	3 (33.3%)
Six	4 (44.4%)

### Procedures

#### Recruitment, eligibility assessment, and enrollment

From February through May 2021, eligible mothers of newborns hospitalized in a Level 4 NICU of a midwestern academic hospital learned about the study in a phone call from the nurse recruiter and subsequently through a recruitment flier in their newborns admission folder. In June 2021, because of low enrollment and because Zoom delivery of Listening Visits allowed women to participate from a distance, recruitment expanded to users of a mobile application targeting new parents through a brief advertisement for the study which appeared on the app’s home page. Women interested in learning more about the study clicked on the ad to read a more extended description. Because the nurses might connect with women in other states, the advertisement was restricted to appear in states in which the Listening Visits nurses had reciprocal nursing licensing agreements.

Across recruitment sites potential participants completed a REDCap survey comprised of screening questions to determine whether they were demographically and clinical eligible to participate. Women were first presented with three demographic screening questions: their age, whether they were currently receiving counseling, and whether their newborn currently hospitalized. If they did not meet all three of these criteria, they received a programmed message indicating reason for ineligibility. Women who met these criteria were then presented with the EPDS items (24). Women were clinically eligible to participate in the open trial if they had mildly to moderately severe depression symptoms, defined as an EPDS score of score >12 and <20 (25) and ≤1 on the self-harm item. Eligible women who were interested in enrolling into the open trial completed informed consent by reading the informed consent document online and signing. Women who were not eligible because their EPDS scores were in the severely depressed range or who were had ratings of 2 or 3 on the self-harm item, received a message explaining that they appeared to be experiencing a level of emotional distress warranting support from a mental health specialist. A tollfree number to two websites listing therapist nationwide and a national crisis hotline were provided. For local women, the number to a women’s wellness specialty clinic was also provided.

#### Intervention

Listening Visits were delivered by three NICU RNs who had completed Listening Visit training requirements and who had previously provided in-person Listening Visits for our prior RCT evaluation, and found to deliver the visits with high fidelity (23). In consultation with IT, the nurses participated in a brief training in how to conduct a secure Zoom session. The nurses hosted Zoom Listening Visits outside of working hours, from a private place within their home to ensure confidential sessions. Nurses were compensated from grant funds. Participants could receive up to six, ∼60-min sessions, scheduled at a mutually agreeable time, approximately two sessions per week.

The general structure of a single Listening Visits session entails greeting, debriefing about the previous visit, updating on current issues, working on a specific issue through reflective listening and problem solving, and providing closure through summarizing the work of the visit. As a non-directive treatment, the woman determines the focus of each session: typically, the first two sessions might focus on the participant’s birth experience, followed by a focus on how the baby is doing and the mother’s concerns and needs. In the last session, the NICU nurse typically provides closure by reviewing progress and evaluating the need for additional mental health services (26).

#### Assessments

To complete the self-report measures that comprised the study assessments, participants received a link to REDCap survey via email (27). Women completed the baseline assessment immediately after enrolling into the study. At 4- and 8-weeks post enrollment, they received an email with a link to a REDCap survey. Compensation checks were mailed to participants: $50.00 for the enrollment and 4-week assessment and $25.00 for the briefer 8-week assessment.

#### Clinical safety, zoom privacy and data security

Women with symptoms in the severely depressed (EPDS score was 20 or above: (25) or who had a rating of 2 or 3 on EPDS self-harm item were not eligible to participate in the trial. The Listening Visits nurses also had access to the hospital’s specialty perinatal mental health clinic and to the Department of Psychiatry. External oversight was provided external reviewers in biannual meetings of the data and safety monitoring committee. In alignment with guidance from the University’s IT Security and Policy Office, nurses utilized the University’s Zoom account to create the meeting. This account has the Zoom passcode automatically set. Additionally, in alignment with the IT Zoom policy, nurses created a unique ID for each meeting and shared the link privately in an email. Zoom does not allow recording of the sessions without notifying all participants. While the nurses did not record the Listening Visits sessions, it was possible that women could have used a device to record their session. Data were stored electronically in REDCap in which primary data and data backups are secured in two separate data centers. Operating system security included secure logins, remote system logging and configuration, and change management.

### Variables and measures

#### Demographics

A questionnaire developed for this trial assessed the demographic characteristics of participants including, age, ethnicity, race, marital status, education level, number of paid hours worked per week, and income.

#### Number and timing of Listening Visits

Nurses completed a log indicating the number and dates of each Listening Visits session.

#### Depression symptoms

The Edinburgh Postnatal Depression Scale (EPDS) was used to determine eligibility for this trial. The EPDS is a 10-item instrument developed to assess depressive symptoms in postpartum women; it has also been validated with pregnant women and women with toddlers (24). The cutoff score of 12 or greater used in this study has a sensitivity and a specificity of 0.86 and 0.78, respectively (24).

The Inventory of Depression and Anxiety Symptoms General Depression scale (IDAS-GD) was used to examine changes in depression symptom severity over time (28). Using Likert-rating scales, this 20-item, self-report, depressive symptom scale assesses dysphoria, suicidality, lassitude, insomnia, appetite loss, and well-being. Psychometrically, the scale correlates significantly with the Beck Depression Inventory–II and has demonstrated its validity and reliability as a measure of depression symptoms (28). The internal consistency of the IDAS-GD was high across the three assessments performed during this trial, with Cronbach’s alphas ranging from 0.88 to 0.94.

#### Satisfaction

Women’s satisfaction with telehealth Listening Visits was assessed with a modified version of the Client Satisfaction Questionnaire [CSQ; (29)]. This 8-item self-report scale assesses perceived effectiveness and satisfaction with treatment. Using a 4-point Likert scale, women rated seven aspects of their treatment: the quality of the intervention; the degree to which the intervention met their expectations (2 items); amount of help provided; their satisfaction with the help received; their assessment of the effectiveness of the help; and willingness to receive it again or recommend it to a friend. For the current study, the wording was modified by substituting “Listening Visits” for “treatment.” The CSQ has previously demonstrated a high degree of internal consistency and correlates well with other estimates of satisfaction (29). Internal consistency of the CSQ was high. Cronbach’s alpha of the CSQ in the current trial was 0.88.

### Data analyses

Depression outcomes were tested using intention-to-treat (ITT) analyses of the final sample (*N* = 9; see Figure 1). Changes over time in IDAS-GD scores were tested using a mixed model analysis with treatment dosage included as a covariate; however, its inclusion had negligible effect on results, so only unadjusted results are shown. The CSQ were analyzed using descriptive statistics. Analyses were conducted in SPSS 28.0.

## Results

### Feasibility

Three NICU nurses were able to provide Listening Visits over Zoom to nine participants. Women completed an average of 4.6 sessions (SD = 1.5; range 2–6). As indicated in Table 1, 44% of participants completed the full course of six sessions. In terms of timing, three participants completed six sessions within 1 month, indicating that they were successful in meeting two to three times per week (Table 1).

### Effectiveness

The effect of time on depression from the mixed model analysis of IDAS-GD was statistically significant (*F*(2,26) = 10.50, *p* < 0.001), indicating improvement in IDAS-GD scores from the baseline to 8-week assessments. Paired-sample *t*-tests of mean differences between specific time points suggested large effect sizes for a decline in symptoms from pre- to post-Listening Visits and from pre-Listening Visits to follow-up (Table 2).

**TABLE 2:** Mean differences in Inventory Depression and Anxiety Symptoms-General Depression scale (IDAS-GD).

Pre-LV		Post-LV		Effect size *d* (pre to post)	Paired *t*-test	df	*P*-value	Follow-up		Effect size *d* (pre to follow-up)	Paired *t*-test	df	*P*-value
Mean	SD	Mean	SD					Mean	SD				
56.89	11.85	40.11	13.78	1.03	3.09	8	<0.015	32.39	10.04	2.08	5.89	7	<0.001

IDAS-GD, Inventory of Depression and Anxiety Symptoms General Depression scale.

### Satisfaction

Eight of the nine participants completed the CSQ at the 8-week assessment, thus providing data pertaining to acceptability of the intervention. The average CSQ score was 29.0 (SD = 3.3), comparable to levels of satisfaction reported by clients receiving depression treatment from a mental health professional (30): 75% of the participants rated the quality of help they received as “excellent,” and 87.5% indicated that they would recommend Listening Visits to a friend.

## Discussion

In this pilot feasibility trial, we learned that it possible for NICU nurses to provide Listening Visits over Zoom. None of the nurses had prior experience with Zoom, and each acquired this skill after one individual coaching session, and no one experienced technological issues in connecting with participants. Initially, the nurses – who had all provided in person Listening Visits in the RCT – missed the direct contact with mothers. However, they quickly reported that women valued the time to talk, especially during the pandemic when so many people experienced social isolation. They also reported that having the sessions over Zoom made it easy to reschedule visits that were canceled at the last minute. With regard to timing of the sessions, three women receiving Listening Visits over Zoom completed all six sessions within 1 month, indicating that they had followed the 2–3 times per week schedule. The ability to provide Listening Visits to mothers via Zoom may be advantageous from an early-intervention perspective. Finally, the use of Zoom allowed the NICU nurses to provide the visits to women living in other states with reciprocal nursing licensure agreement with the host state. Note however, that while there were no issues working with women from other states, the nurses expressed a preference for working with in-state women because of their own greater familiarity with available resources.

With regard to effectiveness of Listening Visits delivered by a nurse over Zoom, the small sample of women in this pilot realized a significant pre to post treatment decrease in depression symptoms. This preliminary finding aligns with the substantial empirical support for in-person Listening Visits (20) as well as evidence indicating that synchronous telehealth modality is as effective as psychotherapy delivered in person (31). In addition to reporting a significant decrease in depression symptoms across the trial, participants also reported high satisfaction with the intervention.

### Limitations

In conducting this small, pilot trial, we had the opportunity to assess the feasibility of providing Listening Visits via Zoom. Yet the design of this trial was by nature expedient, the result of special circumstances: i.e., pandemic restrictions, unused funding from a halted RCT. The small, non-representative sample, the absence of a control group comparator, the absence of fidelity assessments, and the focus on only depression symptoms when anxiety is also prevalent in this population of women (32, 33), limit drawing definitive conclusions about the effectiveness of telehealth Listening Visits. Although participants’ ratings of satisfaction with Listening Visits were high, only nine of 79 eligible women enrolled into the trial. This low enrollment rate may indicate a lack of interest in receiving Listening Visits. Alternatively, it may reflect that during the pandemic women with hospitalized newborns were overwhelmed both in terms of the lifestyle changes generally and the restrictions specific to visiting in the NICU. Additionally, the low enrollment may be attributable to either reluctance to participate in a research study or fatigue with the online enrollment procedure that required women to independently navigate the eligibility assessment and long informed consent document. To more accurately understand women’s willingness to utilize this form of support, it will be critical to disaggregate receipt of Listening Visits from participation in a research trial.

## Conclusion and future directions

Listening Visits delivered over Zoom by NICU nurses provides emotionally distressed mothers with an accessible and convenient option for receiving support during a stressful period. Among the general population of perinatal women, receiving telehealth visits are a valued and convenient alternative to in-person sessions (34, 35), obviating logistical challenges including, but not limited to, finding time, transportation, and childcare. With the evidence for Listening Visits established (20), and proof-of-concept demonstrated for Zoom delivery, the time has come to move this evidence-based approach into clinical care in the NICU setting. The challenge is that Listening Visits, like a multitude of other evidence-based practice (EBP) interventions, may still fail to be translated into clinical practice despite strong positive empirical evidence. In fact, records suggest it takes an average of 15 years to translate research to practice (36). Although evidence for Listening Visits is strong, a number of practical issues still need to be addressed to bridge the gap between research and clinical use. For example, in this post-pandemic era, it is not clear whether telehealth Listening Visits are the preferred meeting option. In the NICU setting, nurses’ time to provide Listening Visits has always been compensated by grant funding. To address these key implementation issues, an important next step in this program of research will be to collaborate with a team of key NICU stakeholders, including but not limited to NICU parents, staff nurses, nurse managers, hospital administrators, social workers, to design a plan of how to integrate Listening Visits into the standard clinical care provided to parents of hospitalized newborns.

## Data availability statement

The raw data supporting the conclusions of this article will be made available by the authors, without undue reservation.

## Ethics statement

The studies involving human participants were reviewed and approved by University of Iowa Institutional Review Board. The patients/participants provided their written informed consent to participate in this study.

## Author contributions

LS and RCD: study design, data collection, manuscript development, and revisions. JM and SA: data analysis, manuscript development, and revisions. All authors contributed to the article and approved the submitted version.
